# Causes of delayed antenatal care at an urban community health centre in Gauteng, South Africa

**DOI:** 10.4102/safp.v67i1.6093

**Published:** 2025-04-23

**Authors:** Siphesihle P. Mlambo, Ozoemena J. Ibeziako

**Affiliations:** 1 Department of Family Medicine, Faculty of Health Studies, University of Pretoria, Pretoria, South Africa

**Keywords:** antenatal care, late antenatal care, basic antenatal care, basic antenatal care plus, pregnant woman, Tembisa clinics, maternal health, South Africa

## Abstract

**Background:**

Maternal and perinatal deaths remain significant despite various strategies that have been implemented. Antenatal care (ANC) for pregnant women is crucial in reducing maternal and child mortality. Delayed ANC is associated with several maternal and foetal complications, which can be prevented through timely intervention. Previous studies have identified various factors contributing to the late initiation of ANC, and although recommendations have been made and implemented, there has been no impact. Objectives were to determine and rank the factors contributing to the delayed initiation of ANC among pregnant women attending a community health centre in the Tembisa township and to explore potential strategies for the early initiation of ANC.

**Methods:**

A cross-sectional survey was conducted using a validated questionnaire on pregnant women attending their first ANC after 20 weeks of gestation.

**Results:**

Multiple variables affecting the early initiation of ANC were identified, namely healthcare workers’ behaviour, negative attitude, operational management factors and participants’ socio-economic standing. Staff counselling, support and training in holistic maternal healthcare, as well as accurate, uniform and consistent health educational information that recognises and addresses cultural beliefs, could encourage early initiation of ANC.

**Conclusion:**

Staff empowerment and support for maternal health care through wellness services are crucial. Clinic operational management should adopt best practices to address prolonged time spent accessing services. Relevant health educational information for change should be provided.

**Contribution:**

The study‘s findings offer insights into the factors that delay the timely initiation of ANC and strategies to mitigate these delays.

## Introduction

Late initiation of antenatal care (ANC) has had a significant negative impact on maternal and child health outcomes. World Health Organization (WHO) has defined delayed initiation of ANC as ‘first hospital or clinic antenatal attendance at 20 weeks or more gestation’.^1^ World Health Organization ANC guidelines released in 2016 showed that globally, only 64% of women received ANC four or more times throughout their pregnancy.^1^ The Saving Mothers report, South Africa (2011–2013) indicated that 16.6% of women who died during pregnancy did not attend the ANC clinic, and 7% attended occasionally.^2^ These results suggest that a gap remains in enhancing the interactions between pregnant women and healthcare providers. The sixth report of the National Committee for the Confidential Enquires into Maternal Deaths (NCCEMD) indicated that 65% of maternal deaths were attributed to avoidable factors such as hypertension, infections and bleeding.^3^ WHO ANC guidelines demonstrated that as the milestone to eliminate mother-to-child transmission (MTCT) for HIV is tackled, there is a rising challenge of other transmittable diseases from mother to child, such as hepatitis, malaria, syphilis, etc., which could be avoided with early initiation of ANC because they could be prevented, detected early or treated.^4^ How to address these challenges is provided in the guidelines, and one of the recommendations is strengthening antenatal and postnatal care for both HIV-negative and -positive mothers.^4^

The perinatal period is an essential indicator of the health status of pregnant women, foetuses and newborns.^5^ Thus, maternal and perinatal mortality data are vital in providing critical information on outcomes that need to be improved to reduce perinatal and maternal deaths.^3,5^ Perinatal mortality is also the best marker of the quality of care offered to the pregnant woman during pregnancy and post-partum.^5^ In 2012, South Africa adopted the National Development Plan (NDP) with the proposed outcome of a long and healthy life for all.^5^ The NDP for South Africa acknowledged that reducing infant and maternal mortality is vital in driving a healthy and long-life narrative for all. However, while high-income countries are succeeding in the fight, South Africa, a middle-income nation, continues to grapple with the tragedy of high maternal and perinatal mortality rates.

The 2016 National Department of Health (NDoH) Maternal Guidelines stated that a woman should visit her healthcare provider when she suspects pregnancy, even as early as the first missed menstrual period.^6^ Several studies^7,8,9,10^ on factors contributing to the late initiation of ANC have been conducted in South Africa. Recommendations have been made, and some have been implemented. Despite this, clinicians at the community health centre serving the Tembisa township continue to observe a pattern of late ANC initiation.

The study aimed to determine factors contributing to late ANC initiation among pregnant women attending antenatal clinics in Tembisa. The objectives include describing the demographics of the women who initiated ANC late, determining and ranking the factors contributing to late ANC initiation and eliciting from participants strategies that could encourage early ANC initiation.

## Research methods and design

### Study design

The study was a descriptive cross-sectional survey to determine the factors contributing to late antenatal initiation.

### Study setting

The survey was conducted at the Ebony Park Community Health Care Centre (CHC), which borders the Ekurhuleni Health District and the City of Johannesburg. Because of its location, most patients who utilise its services reside within the Tembisa township. The CHC began operating as a 24-h facility in 2020. Reports from the obstetrics unit, which supports its maternal health services, indicated that many pregnant women initiate their first ANC visit late. These reasons informed its selection as the study site for this survey.

### Study population and sampling strategy

Using a convenience sampling method,^11^ consenting pregnant women who booked after 20 weeks’ gestation for ANC were recruited. Those who booked before 20 weeks were excluded from the study. Convenience sampling was used to attain the targeted number of participants and facilitate sampling. All walk-ins and booked patients who met the inclusion criteria and signed a written consent on the day of data collection were enrolled in the study.

The sample size was determined using the Clopper-Pearson^12,13^ exact method formula (Equation 1):


$$\left(N=1.96^{2}\times p\times (1-p)/d^{2}\right)$$ [Eqn 1]

where N is the required sample size, p is the proportion of the expected outcome (0.5) and *d* is the precision required (0.16). A sample size of 150 participants produced a two-sided 95% confidence interval (CI). As many categorical variables were estimated in this study, we used a generic prevalence of 0.5 or 50% as the sample prevalence to produce the most conservative (widest) CI width according to the binomial distribution.

### Data collection

A directly administered questionnaire was used for data collection. The researcher designed the questionnaire in English because it is the common language among the diverse cultural groups in the study setting. The questionnaire was piloted to ascertain its validity. The researcher administered the questionnaire and provided oral translations where necessary. Data were collected daily from 06 April 2022 to 30 June 2022 to ensure the determined number of participants was attained. Daily data collection took place on weekdays in April 2022 and May 2022, except Tuesdays because of work commitments, and then daily in June 2022 to ensure the determined sample size was attained. The first part of the questionnaire consisted of de-identified participants’ socio-demographic information. The second part was the 24 factors contributing to late antenatal booking from previous studies,^7,8,9,10^ to which participants reflected their level of agreement using a four-point Likert scale, where one meant the least important, and four was the most crucial factor. The last section was an open-ended question to prompt participants’ suggestions on encouraging early ANC initiation.

### Data analysis

A total of 150 questionnaires were distributed, fully completed and analysed. Participants responded to 24 factors: operational management, personal issues, socio-economic, cultural, religious beliefs and healthcare professional factors. These factors and the participants’ socio-demographics were summarised using frequencies and percentages. Participants’ responses have been hierarchised from highest to lowest using the obtained percentages. The higher the percentage response, the more likely the factor influenced the decision to delay ANC initiation. Participants’ suggestions to encourage early ANC initiation were coded after re-reading to recognise patterns, recurring ideas or phrases. The codes were categorised into themes.

### Ethical considerations

Ethical approvals were obtained from the Faculty of Health Sciences Research Ethics Committee of the University of Pretoria (613/2021), the Ekurhuleni research committee (Research project no: 05/11/2021-03) and the National Health Research Database (NHRD no: GP_202110_010).

## Results

### Socio-demographic characteristics

One hundred and fifty pregnant women attending their first ANC visit after 20 weeks gestation in Ebony Park clinic participated in the study. Most participants, 118 (78.7%), were below 35 years old, and only a fifth (*n* = 32, 21.3%) were above 35 years.

Table 1 shows the socio-demographic characteristics of respondents. Half of the respondents (*n* = 76, 50.7%) were single; a smaller proportion were co-habitating (*n* = 42, 28%) and 23 (15.3%) were married. The rest of the participants (*n* = 9, 6%) were either widowed, divorced or separated. Almost all respondents (*n* = 149, 99.3%) had attained some level of education, while less than 1% (*n* = 1, 0.67%), received no formal education. Slightly over half of the participants were unemployed (*n* = 86, 57%), less than a fifth were employed (*n* = 24, 16%) and less than a third (*n* = 40, 27%) lived off piece jobs were self-employed or had other sources of income, such as government grants.

**TABLE 1:** Demographic characteristics of the respondents.

Variable	Response	Frequency (*n*)	%
Age (years)	15–19	15	10.0
	20–24	33	22.0
	25–29	45	30.0
	30–34	25	16.7
	40–44	26	17.3
	> 45	6	4.0
Marital status	Single	76	50.7
	Married	23	15.3
	Widowed	3	2.0
	Divorced	4	2.7
	Co-habitating	42	28
	Separated	2	1.3
Education	Primary	6	4.0
	High school	129	86.0
	Tertiary	14	9.3
	None	1	0.7
Distance to clinic	Walking	96	64.0
	One taxi	49	33.0
	One bus	1	0.6
	Two taxis	1	0.6
	Two buses	3	2.0
	Own car	0	0.0
Occupation	Unemployed	86	57.0
	Self-employed	8	5.0
	Employed	24	16.0
	Piece-jobs	22	15.0
	Others (grant)	10	7.0
Parity	One	27	18.0
	Two	45	30.0
	Three	46	30.7
	Four	30	20.0
	Five or more	2	1.3
Household income	Low (< R10 000.00)	130	86.7
	Middle (R10 000.00 – R25 000.00)	16	10.7
	High (> R25 000.00)	4	2.6

**Total**	**-**	**150**	**100.0**

Over half of the participants (*n* = 96, 64%) reside within a walking distance radius; a third (*n* = 49, 33%) needed at least one taxi as a means of transport and a tiny proportion required two taxis (*n* = 1, 0.6%), one bus (*n* = 1, 0.6%) or more than one bus (*n* = 3, 2%). Household income ranged from R10 000.00 to R25 000.00 per month. Most respondents (*n* = 130, 86.7%) fell within the low-income household category of below R10 000.00 per month, while a tenth (*n* = 16, 10.7%) are middle-class income household earners between R10 000.00 and R25 000.00 per month. Only a minority (*n* = 4, 2.6%) earned above R25 000.00 monthly. Most respondents (*n* = 123, 82%) were multiparous, and less than a fifth (*n* = 27, 18%) were primigravidas.

## Ranking of factors contributing to late antenatal booking

Participants responded to factors that could contribute to late antenatal booking on a Likert scale of 1–4. A Likert scale of one meant the factor was unimportant, and four meant the factor was crucial and might influence the participant’s decision to start or delay the first ANC visit. Table 2 presents the details of participants’ responses per group of factors.

**TABLE 2:** Participants’ responses on factors for late antenatal care booking.

Factors for late antenatal care booking	Do not know		Prefer not to say		Not important		Very important	
	Frequency (*n*)	%	Frequency (*n*)	%	Frequency (*n*)	%	Frequency (*n*)	%
**Operational**								
I waited in a long queue to be seen by a doctor or nurse	10	6.7	12	8.0	33	22.0	95	63.3
We have to come to the clinic too many times.	22	14.7	7	4.7	27	18.0	94	62.7
The services we get in clinics are poor.	14	9.3	17	11.3	41	27.3	78	52.0
**Personal issues**								
I am not sure if I want to keep the baby.	12	8.0	9	6.0	79	52.7	50	33.3
I am avoiding testing for HIV.	2	1.3	1	0.7	123	82.0	24	16.0
I did not see or feel any pregnancy symptoms.	12	8.0	2	1.3	89	59.3	47	31.3
I was afraid my partner would leave me.	2	1.3	3	2.0	87	58.0	58	38.7
I was scared of my parents’ reaction.	3	2.0	3	2.0	115	76.7	29	19.3
**Socio-economic**								
I have no money for transport.	4	2.7	1	0.7	70	46.7	75	50.0
I do not want to lose my job.	2	1.3	6	4.0	122	81.3	20	13.3
I work from morning to evening, with no time for clinic.	3	2.0	6	4.0	115	76.7	26	17.3
I do not have a permanent residential address.	4	2.7	0	0.0	65	43.9	79	53.4
**Cultural values**								
A woman should not travel during pregnancy.	128	85.3	0	0.0	18	12.0	4	2.7
A pregnant woman must not eat certain foods such as ‘oranges because it causes yellow eyes’.	136	90.7	0	0.0	10	6.7	4	2.7
A pregnant woman must eat certain foods, such as leaves from trees because they contain vitamins.	139	92.7	0	0.0	10	6.7	1	0.7
The husband or partner decides whether a woman should go to the clinic or not.	130	86.7	3	2.0	16	10.7	1	0.7
The last-born child must be delivered at home.	38	92.0	0	0.0	11	7.3	1	0.7
**Religious beliefs**								
I believe that prayer may heal everything.	73	48.7	24	16.0	33	22.0	20	13.3
A pregnant woman should not be touched by a male doctor or nurse.	82	54.7	19	12.7	46	30.7	3	2.0
**Healthcare professionals**								
Nurses might hit you.	52	34.7	11	7.3	18	12.0	69	46.0
Nurses might shout or scream at you.	9	7.3	2	0.7	10	6.7	129	86.0
People will know why you are in the clinic.	5	3.3	7	4.7	123	82.0	15	10.0
Fear of being stigmatised because I have HIV.	16	10.7	4	2.7	119	79.3	11	7.3
Fear of being humiliated by healthcare workers.	10	6.0	1	1.3	9	6.0	130	86.4

HIV, human immunodeficiency virus.

### Healthcare worker’s attitude and behaviour

The majority of participants stated that humiliation (*n* = 130, 86.4%) and bad attitudes (*n* = 129, 86%) by healthcare workers (HCWs) played a significant role in delaying attending ANC. Less than half of the participants (*n* = 69, 46%), even feared being physically assaulted by HCW. Only a few were concerned about lack of confidentiality (*n* = 15, 10%) or still fear HIV stigmatisation (*n* = 11, 7.3%).

### Clinic operational management factors

About two-thirds of the participants indicated that the main factor for late ANC booking was long waiting hours (*n* = 95, 63.3%) and multiple clinic visits (*n* = 94, 62.7%). Half of the respondents (*n* = 78, 52%), indicated poor services as decisive for late ANC visits.

### Socio-economic factors

Half of the participants ranked not having a permanent address (*n* = 79, 53.4%) and not having transport fare (*n* = 75, 50%) as very important factors in late ANC booking. Less than a fifth did not want to lose their jobs (*n* = 20, 13.3%), nor had the time (*n* = 26, 17.3%) to book early for ANC.

### Personal factors

A third of respondents cited fear of partner rejection (*n* = 58, 38.7%), ambivalence to keep the pregnancy, 50 (33.3%), and not feeling any pregnancy symptoms, 47 (31.3%), as causes for delay in attending ANC. Less than a fifth of participants mentioned fear of parents’ reaction (*n* = 29, 19.3%) and HIV stigma, 24 (16%), as reasons to delay even revealing their pregnancy status.

### Religious and cultural factors

As shown in Table 2, religious and cultural factors had minimal influence on participants’ decisions on when to start ANC. Participants were mainly ambivalent about religious and cultural values that contributed to late ANC booking.

Table 3 highlights that the behaviour of healthcare professionals, clinic operational management systems and patients’ socio-economic status are decisive factors in delaying ANC.

**TABLE 3:** Hierarchy of very important factors for late antenatal care booking.

Factors for late antenatal booking	Very important factors		
	Frequency (*n*)	%	Hierarchy
**Healthcare professionals behaviour**			
Fear of being humiliated by healthcare workers	130	86.4	1
Nurses might shout or scream at you	129	86.0	2
**Operational management**			
I waited in a long queue to be seen by a doctor or nurse	95	63.3	3
We have to come to the clinic too many times	94	62.7	4
Socio-economic status			
I do not have a permanent residential address	79	53.4	5

Table 4 shows the importance of ANC services to participants. Most respondents felt it was essential to utilise ANC services, while just over half, 79 (53.4%), acknowledged the importance of frequent ANC visits.

**TABLE 4:** Participants’ responses on the importance of antenatal care services.

Antenatal care services (ANC services)	Do not know		Prefer not to say		Not important		Very important	
	Frequency (*n*)	%	Frequency (*n*)	%	Frequency (*n*)	%	Frequency (*n*)	%
To attend the antenatal care clinic	4	2.7	1	0.7	6	4.0	139	92.7
To be taught about breastfeeding and formula feeding while pregnant	1	0.7	0	0.0	5	3.3	144	96.0
To be taught about family planning while pregnant	1	0.7	0	0.0	8	5.3	141	94.0
To be tested for HIV when pregnant	7	4.7	0	0.0	6	4.0	137	91.3
To visit the clinic more than four times when pregnant	65	43.9	0	0.0	4	2.7	79	53.4

ANC, antenatal care; HIV, human immunodeficiency virus.

## Strategies suggested by participants to encourage early antenatal booking

### Staff counselling, support and training

Participants suggested that ANC service providers should undergo psychological evaluations or mental health training to be able to deal with pregnant women. They complained that clinic staff shout and scream at them when they ask for information; hence, they are fearful even to ask questions or come to the clinic, especially when they are not sick:

‘I once asked the nurse why I must come every month for checkup, but I didn’t get an answer. Instead, she said it is not her problem if I come or not come; she even went as far as saying I was being rude for questioning her follow-up date.’ (Participant 74, 37 years old, unemployed)

‘I think nurses have their own problems to deal with because they are always angry and should go for counselling.’ (Participant 13, 28 years old, security guard)

### Lack of information

Among the 150 participants enrolled in the study, 12 said they tried to book early after finding out they were pregnant. Unfortunately, they were turned back from the clinic and were told it was too early to book:

‘We were very happy with my husband when we did a home pregnancy test, and it came back positive. It was our first child. I was only one month pregnant, and I went to clinic the following day to confirm and find out what to do. When I arrived in the clinic, they did a test which was positive, and they asked me about my last menstrual period. After checking something like a wheel the nurse said, “it’s still very early to start clinic, you must come back after two or three months.” I went home and decided to come back when my tummy was showing.’ (Participant 36, 37 years old, cashier)

‘I was having stomach cramps and went to the clinic and found out I was pregnant. I think I was only few weeks pregnant since I have not missed a period. After a nurse checked me, she just said congratulations you are pregnant and told me she will only give me panado for stomachache. She didn’t tell me when I must come back for follow up so I went home and thought I will only go back to clinic once I am sick again.’ (Participant 17, 32 years old, unemployed)

Seven participants needed to be made aware that they were late for booking. One participant believed that ANC starts after 5 months of pregnancy, as she believed the first 4 months must be kept a secret:

‘In my culture, you must not tell people you are pregnant in the first four months, so it’s difficult to start clinic when nobody knows you are pregnant; we only start clinic once stomach is big and showing.’ (Participant 78, 38 years old, unemployed).

Participants suggested mounting billboards or message conveyors in the clinics to emphasise when to start ANC:

‘Why it is not written in a big board by the gates when to start clinic or even played in those TV, we see in clinics.’ (Participant 112, 33 years old, sales assistant)

### Extension of services

Some participants raised issues of antenatal bookings being made only during the day from Monday to Friday rather than after hours or on weekends. Participants suggested that ANC services, including bookings, should be rendered daily as a 24-h service for those who cannot come during weekday work hours:

‘It will be better if pregnant women can attend clinic any day because some of us are working long hours and we are only free after work and on weekends, and our bosses don’t give us time off.’ (Participant 1, 37 years old, general worker)

## Discussion

The survey revealed that the top-ranked factors contributing to the delay in ANC initiation were the behaviour and attitude of HCWs, clinic operational management and socio-economic factors. Studies^7,8,9,10^ conducted throughout South Africa support the diverse nature of these factors.

Healthcare workers’ behaviours and attitudes are multifaceted. Various factors influence the types of behaviour and attitude, including organisational factors (such as lack of staff and resources) and individual factors (such as professionals’ beliefs about maternal age, marital status of a pregnant woman, fatigue and skill deficiencies). Imposing personal beliefs on patients may adversely affect them, infringing on their fundamental human right to adequate healthcare services, protection, quality care and positive health outcomes.^14^

The survey findings indicating that participants fear being physically assaulted by HCWs are supported by Roberts et al.,^14^ which notes that HCWs may even resort to physically assaulting patients to assert control or authority over them. This underscores the need for staff welfare programmes and ongoing in-service training to enhance continuous motivation and behavioural change.

In the Saving Mothers report (2014–2016), the committee summarised its recommendations into five key points: the five Hs (HIV, haemorrhage, hypertension, HCW training and health systems strengthening) as the primary focal points to ensure better pregnancy outcomes.^15^ The report identified HIV as the central issue, emphasising the necessity of adopting a non-judgemental approach.^15^ Ensuring confidentiality and fostering a trusting patient-provider relationship could alleviate patients’ fears of stigmatisation, as demonstrated in this study.

Counselling, support and training for maternity staff in the care of pregnant women, aimed at encouraging the early initiation of ANC, may not be unique to this survey. The 2016 ‘CLEVER’ study,^16^ which comprised a range of multi-component interventions designed to alter the complex interplay of preventable factors contributing to maternal and perinatal mortality and morbidity and deficiencies in clinical governance, was conducted in the Tshwane District. This study improved perinatal mortality and morbidity rates in Midwife Obstetric Units (MOU). These enhancements were attributed to supportive supervision, coaching during handovers and capacity building, which collectively ensured improved clinical performance and reduced risks in the labour ward. By extrapolating from the CLEVER package intervention, barriers to staff performance could be alleviated and best practices could be achieved through targeted and ongoing staff training and support.

The survey also identified factors related to clinic operational management as crucial for delayed ANC initiation. Long queues, extended waiting times and inadequate services lie at the core of operational management failures. A study conducted in KwaZulu-Natal^17^ reached similar conclusions; however, its participants expressed satisfaction with the services, only voicing complaints about prolonged waiting times and queues.

Other factors identified in the survey were socio-economic, highlighting the influence of social determinants on health. It also offers insights into the significant inequalities in health status among individuals, particularly between developed and developing countries, as outlined in the Alma-Ata Declaration.^18^ Moreover, the inability of participants to initiate timely ANC because of migration and absence of permanent addresses aligns with a study conducted in Italy,^19^ which demonstrated that women of higher social status or those with established healthy living conditions are more aware of effective health practices during pregnancy. The fear of job loss and the lack of time to attend the clinic emphasise the necessity to engage and inform other community stakeholders, such as employers, about the fundamental right and importance of quality ANC for pregnant mothers.

Socio-demographic factors such as maternal age, marital status, occupation, education and parity did not correlate with the decision to delay seeking ANC. These findings align with a study conducted in Nigeria,^20^ which indicated that most women postponed initiating ANC, irrespective of their education, social class, parity and age.

Participants suggested a place for appropriate and accurate information sharing. The study by Solarin and Black supported this, noting that patients are turned away because they have come too early for antenatal initiation.^21^ This contradicts the NDoH policy that ANC should commence at the pregnant woman’s first visit to the clinic.^6^

### Limitations

The survey was conducted in one of the CHCs in Tembisa, an urban migrant population. A different picture might be obtained if the study is conducted in rural areas where the unemployment rate is high and the distance travelled to clinics is outside a walking distance radius. Convenience sampling, a non-probability sampling method, was used to attain the desired number of participants. As a result, the research findings might not be generalisable.

### Recommendations

Staff empowerment in maternal health care should be more reflexive and not only technical and knowledge-based. This integrated approach could provide HCWs with psychosocial knowledge and skills. Staff wellness services should be accessible to staff who should be informed of the scope of their services. Ongoing debriefing of staff working under challenging circumstances, such as MOU and antenatal services, could create a platform for staff counselling, support and training.

Clinic operational management could benchmark and adopt best practices to make ANC services accessible to its population.

Health education HCWs provide should aim for accurate, uniform and consistent information. In addition, billboards and message conveyor systems, community health workers’ outreach services and community forums could effectively transmit health messages.

## Conclusion

This survey showed that the key factors contributing to late ANC booking were HCWs behaviour and attitude, clinic operational management and socio-economic factors. Socio-demographic characteristics were not relevant to late ANC initiation.

There is an emphasis on maternity staff training and support, as well as empowerment to take a holistic approach to the care of a pregnant mother. Accurate, uniform and consistent health education should recognise and address cultural beliefs hindering early antenatal initiation.
